# Neural shifts in alpha rhythm's dual functioning during empathy

**DOI:** 10.1002/brb3.2355

**Published:** 2021-09-18

**Authors:** Niloufar Zebarjadi, Jonathan Levy

**Affiliations:** ^1^ Department of Neuroscience and Biomedical Engineering Aalto University Espoo Finland; ^2^ Baruch Ivcher School of Psychology Interdisciplinary Center Herzliya Herzliya Israel

**Keywords:** Alpha rhythm, development, fMRI, MEG, Pain empathy, Social neuroscience

## Abstract

**Introduction:**

Alpha oscillations are unique in their capacity to relay neuronal information through a dual‐process named “gating by inhibition”: rhythmic enhancement inhibits task‐irrelevant regions while rhythmic suppression engages task‐relevant regions in the brain. A social‐cognitive process that operates by relying on the suppression of the alpha rhythm in the primary somatosensory cortex (S1) is the ability to generate empathy. This phenomenon has been evidenced in dozens of electrophysiological studies targeting adult human subjects. Yet, recent studies on the neurodevelopment of empathy indicate that in younger age, empathy does not involve alpha suppression in S1 but only enhancement. More interestingly, right before adulthood, this rhythm is still enhanced, but in a remarkable shift, a pattern of suppression emerges. In this registered magnetoencephalography (MEG) and functional magnetic resonance imaging (fMRI) study, we will capture frequency‐decomposed neural activity particularly at the alpha range and its corresponding hemodynamic response and target subjects at around 20 years old as a unique time‐window in development that allows investigating in parallel both low‐alpha suppression and high‐alpha enhancement. We aim to address two questions: (a) Does alpha power suppression in the S1 region during empathy correspond to BOLD increase in this region? (b) What is the functional role of alpha power enhancement during empathy development (BOLD signal increase or decrease)? Addressing these questions will particularly advance knowledge on the process of empathy in the brain, and the way in which it is underpinned by alpha oscillations. Moreover, examining these experimental outcomes can potentially lay the ground for future studies that would further examine the role of alpha oscillations in empathy during the course of development.

**Methods:**

Brain data of forty healthy individuals close to 20 years old will be recorded in two consecutive MEG and fMRI sessions while subjects observing physical pain versus neutral stimuli. Besides, each participant's subjective experiences wll be measred by questionnaires, interviews and rating of the stimuli.

## Graphical abstract

Weinvestigate here the dual‐process of alpha oscillations. We follow up on our previous surprising studies that found a unique feature of this dual process in the course of the neurodevelopment of empathy. This study will further investigate this phenomenon by implementing within‐subject MEG and fMRI sessions.

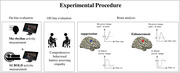

## 1 INTRODUCTION

Empathy is among the most important abilities in human social life: it enables the perception of vicarious emotions and thoughts and is therefore crucial for healthy social interaction. Deficits in empathy can result in aggression, violence, or apathy and can be observed in several psychopathologies (Decety, 2010). Empathy for pain is one of the most basic forms of empathy, sculpted by the long history of mammalian evolution while enhancing species survival and social living (de Waal & Preston, 2017). There have been plenty of studies on neural basis of pain empathy in the brain. Although, similar to other cognitive and social tasks, these studies measured brain response to vicarious pain and not pure empathy, and therefore the effect might be entailed other cognitive processes such as attention or negative effects. These studies typically measured brain oscillation while participants were observing painful and nonpainful stimuli (Cheng et al., 2008; Jackson et al., 2005; Jackson et al., 2006; Lamm et al., 2011; Levy et al., 2016, 2018; Mu et al., 2008; Whitmarsh et al., 2011). One of the pioneering studies on the neural substrates involved in empathy for pain reported the causal role of the primary somatosensory cortex (S1) (Avenanti et al., 2005). This finding was in line with earlier evidence of S1 activation during pain perception (Bushnell et al., 1999) and generated many studies investigating the overlap or dissociation between pain empathy and pain perception (Zaki et al., 2016). In parallel, Electroencephalography (EEG) evidence accumulated to point out that during pain empathy, the sensorimotor cortex and possibly neighboring regions consistently generate oscillation in the alpha‐band (Chen et al., 2012; DiGirolamo et al., 2019; Hoenen et al., 2015; Mu et al., 2008; Peled‐Avron et al., 2018; Perry et al., 2010; Riečanský & Lamm, 2019; Woodruff & Klein, 2013; Woodruff & Maaske, 2010; Woodruff et al., 2011; Yang et al., 2009). However, it was not until that magnetoencephalography (MEG) was used, with its excellent ability to localize rhythmic generators at the surface of the brain, when it became clearer that empathy does not suppress the dominant alpha oscillations that are generated by the occipital cortex. Instead, empathy was found to suppress the mu rhythm (Cheng et al., 2008; Levy et al., 2016; Motoyama et al., 2017; Whitmarsh et al., 2011) (i.e., alpha‐band activity generated in S1 (Salenius et al., 1997)). This MEG evidence was in line with functional magnetic resonance imaging (fMRI) studies reporting BOLD response in S1 following pain empathy (Lamm et al., 2011), as well as with the Transcranial magnetic stimulation (TMS) pointing out causality in pain empathy (Avenanti et al., 2005) and in prosocial behavior (Gallo et al., 2018). This corroborated rich prior evidence on the correlation between suppression of the alpha rhythm and BOLD activation (Scheeringa & Fries, 2019). Altogether, this and other rich literature during the past two decades (DiGirolamo et al., 2019; Hoenen et al., 2015; Joyal et al., 2018; Motoyama et al., 2017; Peled‐Avron et al., 2018; Riečanský & Lamm, 2019; Woodruff & Klein, 2013; Woodruff & Maaske, 2010; Woodruff et al., 2011) point out that alpha rhythm generated by S1 (i.e., mu rhythm) is perhaps the most consistently observed neural representation underlying empathy for others' physical pain.

Despite this apparently undisputable effect, one noteworthy detail raises caution: almost the entire literature on this topic targeted adult subjects. In fact, perhaps the only EEG study that targeted children found no mu rhythm effect during empathy (Cheng et al., 2014), although the study did not further investigate this null effect. Another EEG study on subjects with mean age of about 20 reported alpha power enhancement during pain empathy (Mu et al., 2008). A recent large‐sample MEG study comprehensively addressed this topic by cross‐sectionally sampling 210 adults, 16–18 years old participants and children, and by investigating their rhythmic activity patterns and its cortical generators. The study found surprising developmental effects that drastically shape the alpha rhythm during pain empathy: alpha enhancement in childhood, while both (low‐alpha) suppression and (concurrent high‐alpha) enhancement at the age around 16–18 years, and only alpha suppression in adulthood (Levy et al., 2018). In other words, pain empathy in children mostly relies on sensory alpha enhancement which possibly reflects self‐based sensory processing, develops through a long process of maturation, and finally shifts to alpha suppression (and other higher frequency patterns) in adulthood which perhaps reflects other‐centered processing of pain empathy (Levy et al., 2018). It is important to note that developmental studies on mu rhythm consider upper (10–13 Hz) and lower (6–9 Hz) mu rhythms separately as they demonstrated to have different functional properties (Pfurtscheller et al., 2000; Soroko et al., 2014; Thorpe et al., 2016). This indicated that the mu rhythm effect that has been observed in dozens of neuroimaging studies on empathy reflected an effect only during the mature state of empathy and that robust mechanistic shifts in alpha rhythmicity may reflect the developmental maturation of empathy. Plenty of studies on human and nonhuman primates reported a close connection between neuronal activity and cortical networks’ development and maturation (Uhlhaas & Singer, 2010) and a stronger inter‐regional correlation in the alpha‐band by getting older (Schäfer et al., 2014). For instance, a recent developmental study on the mirror system reported a significant increase in alpha‐band desynchronization by age as well as an increase in the level of empathy by getting older (Brunsdon et al., 2019). Besides, studies on children and adults confirmed the effect and further pointed out that these shifts in alpha rhythmicity are not constrained to pain empathy and also other sorts of empathy, namely affective and cognitive empathy (Levy, Goldstein, et al., 2019; Levy et al., 2019a). To our knowledge, there is not any other study on the other forms on empathy on adolescents or young adults around 20 years. Despite these prominent and surprising effects, several points remained unanswered regarding these rhythmic shifts: What is the functional role of shifting from sensory alpha enhancement to alpha suppression? Does the first reflect inhibition of one cortical patch that gradually becomes active at a later phase in development?

To gain a deeper understanding of this outstanding phenomenon, it is noteworthy that alpha‐band activity is the most dominant rhythm in the awake human brain and has been studied for almost a century (Berger, 1929). These studies pointed out a dual representation of this rhythm, and about a decade ago this mechanism has been proposed to get information throughout the brain (see Jensen & Mazaheri, 2010 for the “gating by inhibition hypothesis”): the suppression in alpha activity in a selective brain region reflects engagement and processing (Bauer et al., 2006; Berger, 1929; Pfurtscheller & Silva, 1999; Van Dijk et al., 2008), whereas the enhancement of alpha activity has been repeatedly shown to inhibit task‐irrelevant regions. Studies on attention, perception, memory, sensory, and motor functioning clearly demonstrated that alpha power enhancement in task‐irrelevant regions mirrors the functional disengagement of these regions (Bauer et al., 2014; Haegens et al., 2010; Mazaheri et al., 2014; Mazaheri et al., 2009; Van Dijk et al., 2010). For instance, during attentional tasks, alpha activity is enhanced in the hemisphere ipsilateral to the attended hemifield, while it is suppressed in the contralateral hemisphere (Bauer et al., 2014). Similarly, during sensory and motor functioning, although alpha suppression is bilateral in somatosensory cortex, hemisphere contralateral to the task side displays significantly greater alpha power suppression (Bauer et al., 2006; Yuan et al., 2010). Likewise, during a working memory task, alpha activity is enhanced in disengaged posterior region and suppressed in the engaged somatosensory region (Haegens et al., 2010; Jokisch & Jensen, 2007). Interestingly, notwithstanding the prominence of alpha in the generation of empathy, its dual‐faceted representation has not been reported thus far in empathy studies. The recent evidence of a developmental shift from enhancement through enhancement‐suppression to suppression of alpha activity during empathy (Levy et al., 2018, 2019b; Levy, Goldstein, et al., 2019) suggest a novel and perhaps unique pattern that may shed light on the dual‐functioning of alpha‐band activity. Importantly, these recent serendipitous findings highlighted age around 20 as a unique time‐window in development that allows investigating in parallel both alpha suppression and alpha enhancement.

One experimental strategy for further investigating this unique mechanistic shift (i.e., enhancement to suppression) in alpha oscillations is by resorting to consecutive sessions of fMRI and MEG. Previous studies found that crossing MEG data with fMRI (measuring BOLD signal) can be informative in obtaining a more comprehensive outlook on brain activity, particularly by capturing frequency‐decomposed neural activity and hemodynamic response throughout the cortex (Dymond et al., 2014; Jensen & Mazaheri, 2010; Kujala et al., 2014; Mathiak et al., 2011). First, while MEG's ability to localize cortical sources is good, it is limited by its reliance on inverse modeling (Gross et al., 2001), whereas BOLD estimation in fMRI offers an excellent spatial resolution. Hence, MEG can straightforwardly measure alpha suppression and enhancement, and fMRI enables to localize the exact BOLD‐activated (engaged) and ‐deactivated (disengaged) brain regions associated with these alpha patterns (Jensen & Mazaheri, 2010; Pfurtscheller et al., 1996; Zumer et al., 2010; Zumer et al., 2014). Second, while previous MEG‐fMRI studies reported the association between alpha suppression and increase in BOLD activity during cognitive tasks (Singh et al., 2002; Yamagishi et al., 2005; Zumer et al., 2010) and between alpha enhancement and BOLD deactivation (Moosmann et al., 2003; Zumer et al., 2014), a study on the relation of BOLD deactivation and neuronal activity suggested that BOLD deactivation is not always associated with neural inhibition (Hayes & Huxtable, 2012). Thus, the combination of electrophysiological and hemodynamic measurements can straightforwardly probe the functionality of the mechanistic shift (i.e., enhancement to suppression) in sensory alpha oscillations, as detailed in the hypotheses below.

**FIGURE 1:**
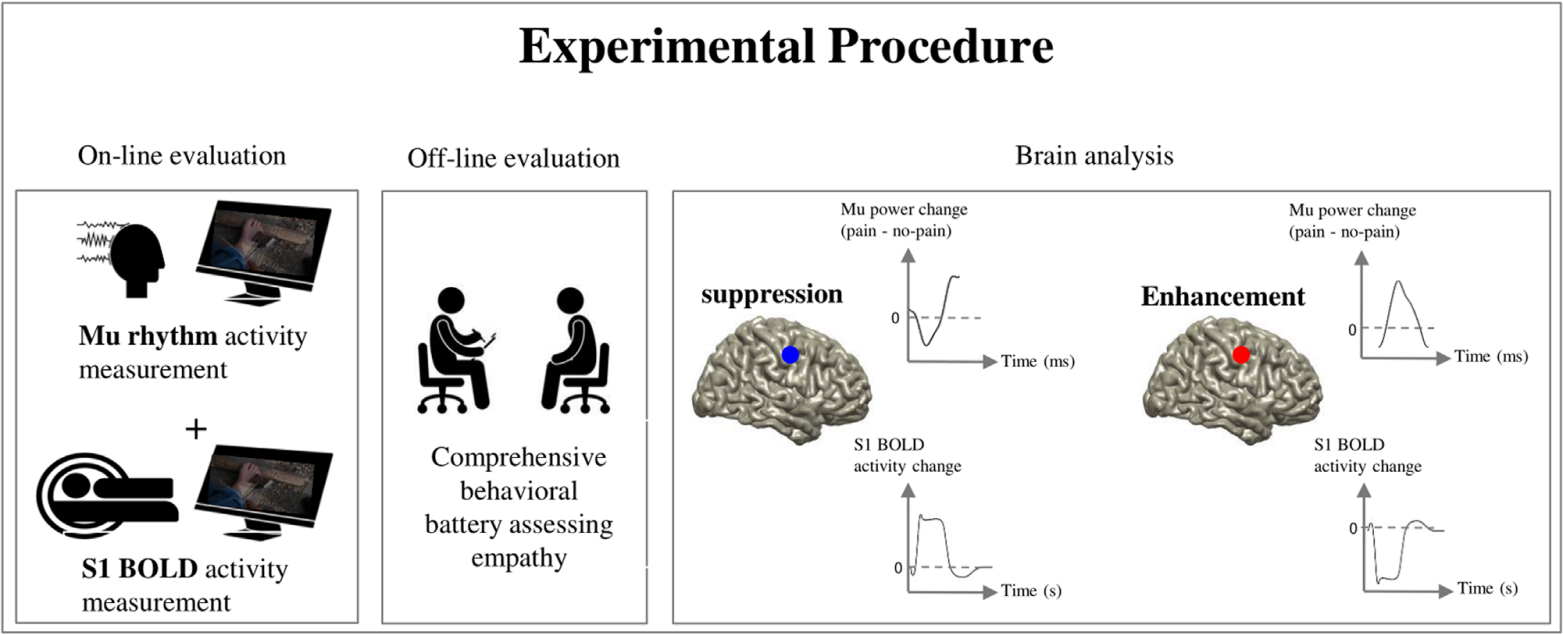
Experimental procedure. Participants will go through a battery of behavioral measures of empathy, after the neuroimaging sessions. During the two sessions (magnetoencephalography (MEG) and then functional magnetic resonance imaging (fMRI)), participants will perceive empathy‐evoking stimuli. The analysis of the neural data obtained from the two instruments will be assessed in parallel to test the hypotheses regarding shifts in the alpha rhythm.

In this combined MEG and fMRI study (Figure 1), we exploit the possibly unique dual‐alpha pattern of empathy at around 20 years old age in order to shed light on the functioning of the alpha rhythm. We measure alpha rhythm and BOLD activity during pain empathy in subjects who are at the age of about 20 years, as past work clearly pointed out that at this stage of development, pain empathy triggers in parallel both alpha suppression and alpha enhancement (Levy et al., 2018); we conducted a brief pilot, which supported this assumption. Then, we will conduct MEG source reconstruction for each of the two neural activity patterns (suppression and enhancement) separately and examine the fMRI BOLD signal in these sources. We formulate two questions: (1) Does the alpha suppression (that is reported in many empathy studies) correspond to BOLD increase in S1 during empathy? (2) What is the relation between alpha enhancement, which has been recently observed in developmental empathy studies, and the BOLD signal during empathy? In other words, in the second question we will explore whether alpha enhancement corresponds to (a) BOLD decrease or (b) BOLD increase in S1 or alternatively in a different cortical patch. Addressing these questions will particularly advance knowledge on the process of empathy in the brain, and the way that it is sustained by alpha oscillations, and potentially lay the ground for future studies that would further examine the role of alpha oscillations in empathy during the course of development. Finally, we assess social and empathy abilities with vicarious pain questionnaire (VPQ) (Grice‐Jackson et al., 2017), interpersonal reactivity index (IRI) (Davis, 1983) questionnaires and by rating of stimuli's perceived pain to explore the potential contribution of inter‐individual differences to the neural effects investigated here.

## 2 METHODS

### 2.1 Participants

Forty healthy individuals, all university students around 20 years old participated in MEG and fMRI study; data acquisition ceased after completion of the 40th subject. Acquired data were excluded from analysis only under circumstances of excessive head movement (which was continuously monitored by the two imaging facilities) or lack of compliance to the specifications in the experimental task. Noteworthy, although we generally conducted very high‐sample MEG studies (e.g., Levy et al., 2018), in this proposed study there was no need to exceed 20–30 subjects as the enhancement/suppression effect was very strong and could be seen in smaller samples (e.g., Cheng et al., 2008; Motoyama et al., 2017; Whitmarsh et al., 2011), or even in only two pilot subjects, as reported below. Nevertheless, we conducted an a priori power analysis based on our previous study (Levy et al., 2016) (*d* = 1.59 and 0.61, for alpha suppression vs. baseline and alpha enhancement versus baseline, respectively), indicating that sample sizes of six and 25 would be sufficient to detect statistically significant alpha suppression and enhancement, respectively, at 90% power. Furthermore, previous fMRI studies on pain empathy reviewed in Lamm et al. (2011) observed the effect with sample size under 25 and only one study had greater sample size. Yet, to maximize the statistical power and reliability of the expected findings in this registered study, we oversampled to 40 individuals, who repeated the experiment twice, in the two neuroimaging instruments.

Participants were right‐handed and asked to fill a primary online survey about history of psychiatric and neurological disorders, MEG/fMRI compatibility and their demographics (such as gender, age, and education level). All participants read an information sheet and a privacy notice paper and signed the participation confirmation form, all approved by the Aalto University Research Ethics Committee.

### 2.2 Experimental design

In both MEG and fMRI experiments, the same task design was applied for increasing reliability in aligning data obtained from the two techniques. Participants were familiarized with the scanning procedures and asked to avoid bodily movements during the scans. The stimuli and their design were similar to several of our previous experiments (Levy et al., 2016, 2018, 2019a; Pratt et al., 2016), that is, 108 images half containing physical pain such as injuries or wounds in the body and half neutral condition (18 action‐images) were presented on a gray background to the subjects. The other half were identical images except of a minor change that would result in conveying no‐pain. Besides, 18 additional control images of simple landscape were presented to the subject to measure still‐images versus action‐images contrast to confirm that suppression and enhancement of alpha oscillation in S1 was unique to empathy. As elaborated in Section 1, the contrast between pain and no‐pain typically evoked sensory alpha‐band activity. Block design was used to maximize detection of BOLD signal. Stimuli were grouped into 42 blocks of three same‐type stimuli. There were 3–3.5 s (random jitters) inter‐stimulus intervals and about 15 s intervals between the blocks. An attentional filler (random twirl) was applied to this semi‐passive task as is often done in this task (Levy et al., 2018).

### 2.3 Data acquisition and preliminary analysis

MEG: During the MEG measurement, the participant sited in the relax position inside the MEG scanner, in front of a screen that presented stimuli to him/her. Stimuli were presented using Presentation software (Presentation; Neurobehavioral Systems Inc., Berkeley, CA, USA). Brain activity was recorded by a whole‐head 306‐channel neuro‐magnetometer (VectorView, Elekta‐Neuromag, Helsinki, Finland) of the MEG Core of Aalto NeuroImaging infrastructure at Aalto University. The MEG device was situated inside a magnetically shielded room equipped with an active noise cancellation system and three‐layer covers to reduce outside magnetic fields. The locations of coils attached to the scalp were recorded for each subject. Five head position indicator (HPI) coils were used and continuous HPI was applied. Eye blinks and saccades were recorded by electro‐oculogram (EOG) electrodes. The data were sampled at a rate of 1000 Hz. During the measuring, a high pass filter of 0.1 Hz and a low pass filter of 330 Hz were applied. MEG data were filtered using Max‐Filter software (Elekta Neuromag) to attenuate measurement artifacts and magnetic interference (from inside and outside of the sensor array) as well as transform data due to head movements. Further MEG data preprocessing was done using MNE‐python toolbox (Gramfort et al., 2013). Raw signal was band‐pass filtered at 1−40 Hz, and eye and heart artifacts were removed during independent component analysis (ICA) by manual detection of these patterns. Events were epoched by aligning the data with the onset of the stimuli and a time window size of −0.5 to 2 was created around the events and bad channels were rejected. To compute time‐frequency representation (TFR), Morlet wavelets method was applied on each trial and average power over epochs was calculated for pain and no‐pain conditions. For plotting, average power in each condition was baseline‐corrected using prestimulus interval of −0.5 to −0.05 s. To conduct source analysis, for each subject, we used a single‐shell brain model based on participants’ anatomical MRI, which was spatially aligned to the MEG sensors and apply beamforming to reveal the cortical sources that generated the activity patterns (low‐alpha suppression and high‐alpha enhancement) separately.

fMRI: MRI data were acquired with a 3 Tesla MRI whole‐body scanner (MAGNETOM Skyra; Siemens Healthcare, Erlangen, Germany) at the Advanced Magnetic Imaging (AMI) Centre of the Aalto University. Participants laid down on a table that slides into the center of the magnet. Subjects saw the screen at 33−35 cm viewing distance via a mirror located above their eyes. Stimuli were presented using Presentation software (Presentation; Neurobehavioral Systems Inc.). The device used a 30‐channel receiving head coil array. The stimuli were presented through AMI Centre's standard setup. Structural images that were acquired with high resolution T1‐weighted (T1w) magnetization prepared rapid gradient echo (MPRAGE) with sagittal orientation, 176 slices, repetition time 2530.0, echo time 3.3, slice thickness 1.00/50%, and base resolution 256. Functional data were acquired by T2*‐weighted echo‐planar imaging (EPI) sequence with axial orientation, 41 slices, repetition time 1000 ms, echo time 32.0, slice thickness 3.1 with a 0% gap, and base resolution 64. fMRI data analysis was performed using MATLAB 2020b and the SPM12 toolbox (www.fil.ion.ucl.ac.uk/spm). Initially, data format was converted to neuroimaging informatics technology initiative (NIFTI) format to be able to process the data with SPM12. Anatomical image was corrected and skull‐stripped. Through a standard fMRI data preprocessing procedure, slice time correction was performed on functional brain images, followed by movement correction and spatial smoothing which was done (8 mm full width at half maximum Gaussian kernel) on the motion corrected data to improve signal‐to‐noise ratio. At the last preprocessing step, the functional images and anatomical MRI image were co‐registered. To generate pain > no‐pain and no‐pain > pain contrast images, first‐level general linear model (GLM) based analysis was conducted. Subsequently, second‐level ANOVA model on whole brain was implemented, and activated and less activated brain regions were selected. In addition, region of interest analysis was performed and average beta estimate of each voxel in the selected regions was calculated to test the differences between pain and no‐pain conditions.

### 2.4 Planned statistical analysis

Our two a priori statistical tests were first related to the MEG and then to the fMRI data: that is, first to test whether pain empathy induced low‐alpha suppression and high‐alpha enhancement. Second, to cross that data with the fMRI data, we source‐localized these two expected effects separately using beamforming techniques, and tested whether they respectively corresponded to BOLD activation and less activation. In other words, the peak coordinates (e.g., (xx1, yy1, zz1)) of the cortical source generating alpha suppression were applied as region of interest in fMRI to test whether it yielded a significant BOLD activation. Based on the literature on alpha response during empathy, we assumed that the coordinates were in S1. We then examined fMRI bold signal in these coordinates. We hypothesized, based on the vast literature matching alpha suppression with bold activation, that S1 alpha suppression would correspond to bold activation. The same was done for the enhancement pattern: if the peak coordinates (e.g., (xx2, yy2, zz2)) of the cortical source generating alpha enhancement were applied as region of interest in fMRI to test whether it yielded a significant BOLD less activation. That might also be generated by S1 based on our previous study (Levy et al., 2016); however, that remained to be determined whether both alpha patterns were generated by the same regions or by distinct substrates. We then repeated these analyses by applying the same approach but originating from fMRI to MEG, that is, peak coordinates of BOLD activation (less activation) were applied as region of interest in MEG to test whether it reflected significant alpha suppression (enhancement). A neutral‐outcome test targeted alpha suppression in the visual cortex. That is, given that visual stimulation triggered robust alpha suppression and BOLD activation in the visual cortex, we contrasted the visual stimuli (e.g., pain and no‐pain pictures) compared to baseline and tested whether the peak coordinates (e.g., (xx3, yy3, zz3)) of the cortical source generating alpha suppression yielded a significant BOLD activation when applied as region of interest for fMRI data. Finally, the MEG statistical tests were conducted versus baseline (i.e., whether alpha power is significantly lower or higher than baseline) and relied on a nonparametric method for multiple‐comparisons correction (Maris, 2007).

### 2.5 Behavioral and self‐reported measurements

After the neuroimaging measurements, in a separate room, stimuli were presented again and participants were required to rate the level of vicarious pain on a 4‐point scale: 1, none; 2, moderate; 3, a lot; 4, extreme after watching each stimulus. It represented how he/she comprehended and felt other's emotional states. In addition, subjects were required to fill questionnaires to evaluate social and empathy abilities. The first questionnaire was VPQ, a qualitative method to measure pain perception (Grice‐Jackson et al., 2017). During VPQ measurement, subjects were asked to watch 16 painful videos and answer questions related to perceiving pain during watching each video (if they answer yes, the level, location, and type of pain are asked). The second one was “empathic concern” and “perspective taking” subscales in IRI for the assessment of trait empathy (Davis, 1983). We expect to see a negative correlation between subjective sensitivity to pain (measuring by VPQ) and the late alpha enhancement that we found in our recent study (Zebarjadi et al., 2021). Besides, we conducted a phenomenological interview to evaluate the subject's social environment and life experiences.

### 2.6 Pilot data

Pilot experiments (*N* = 2) were conducted to validate the first objective: testing the presence of alpha suppression and alpha enhancement in MEG data, and to observe patterns of activation and less activation in the fMRI data. To further test the second objective, that is, testing whether the oscillatory patterns (suppression and enhancement) reflected activation patterns (activation and less activation), a sufficiently large sample size was required, as is planned in this registered study. Although the pilot investigated only two (young adult) subjects, the MEG results are almost identical to those from our previous MEG empathy study wherein alpha power suppression was followed by alpha power enhancement (Levy et al., 2016), even at the single‐subject level. Furthermore, BOLD response in the first subject shows activation in the sensory cortex and less activation in precuneus cortex. In the second subject, activation and less activation are in the superior parietal area. Certainly, many more subjects are required to address the hypotheses raised above, and to test them statistically thereby achieving high statistical power of the expected outcomes. Yet, the pilot results are very encouraging. The scripts used for the pilot analyses are available by request.

### PEER REVIEW

The peer review history for this article is available at https://publons.com/publon/10.1002/brb3.2355.

## Data Availability

The authors agree to register their Stage 1 Protocol in a recognized repository. The authors agree to share their data pending institutional ethical policies.
